# EHMTI-0267. Plasma anandamide concentration after aerobic exercise training in healthy individuals and episodic migraine patients

**DOI:** 10.1186/1129-2377-15-S1-E22

**Published:** 2014-09-18

**Authors:** AB Oliveira, RT Ribeiro, MT Mello, S Tufik, MFP Peres

**Affiliations:** 1 Neurologia and Neurocirurgia, Universidade Federal de São Paulo, São Paulo, Brazil; 2 Psicobiologia, Universidade Federal de São Paulo, São Paulo, Brazil

## Introduction

Anandamide (AEA) is an endocannabinoid operative in several biological functions. Nevertheless, it is not known the effect of aerobic exercise training (EXT) on plasma [AEA].

## Aims

Because a dysfunctional endocannabinoid system has been suggested to underlie migraine (M) pathophysiology, we intended to explore the plasma [AEA] after EXT in M patients and healthy individuals.

## Methods

EXT protocol consisted of 12-week of supervised treadmill at standardized intensity, performed 3 times/week, 30 min./session. Four groups were separated for intervention or waiting list: Healthy subjects without AET (CC), healthy subjects undergoing EXT (CEXE), M patients without EXT (MC), and M patients undergoing EXT (MEXE). Patients had episodic migraine with and without aura (ICHDII). Blood collections were performed interictally at least 24h after attacks or anti-inflammatory use. AEA was quantified by LC/MS/MS. All participants took no preventive medication.

## Results

The study included 48 participants (12 for each group) and groups matched by age, sex, and BMI. Baseline [AEA] was not different between groups. AEA reduced in MEXE and CEXE, but was statistically significant only in CEXE (p = 0.007). There was a trend to a negative correlation between adherence and ΔAEA (r=-0.565, p = 0.056).

## Conclusions

Plasma AEA decreases after EXT in healthy subjects. In M patients, this response is prevented by lower adherence. Future studies should investigate the relationship between exercise-reward and AEA.

* p = 0.007, Friedman´s Test

No conflict of interest.

